# Modern Electrode Technologies for Ion and Molecule Sensing

**DOI:** 10.3390/s20164568

**Published:** 2020-08-14

**Authors:** William S. Skinner, Keat Ghee Ong

**Affiliations:** 1 Department of Chemistry, University of Oregon, Eugene, OR 97403, USA; wss@uoregon.edu; 2 Phil and Penny Knight Campus for Accelerating Scientific Impact, University of Oregon, Eugene, OR 97403, USA

**Keywords:** ion-selective, electrode, electrochemical, voltammetry, environmental sensors, bio-sensors

## Abstract

In high concentrations, ionic species can be toxic in the body, catalyzing unwanted bioreactions, inhibiting enzymes, generating free radicals, in addition to having been associated with diseases like Alzheimer’s and cancer. Although ionic species are ubiquitous in the environment in trace amounts, high concentrations of these metals are often found within industrial and agricultural waste runoff. Therefore, it remains a global interest to develop technologies capable of quickly and accurately detecting trace levels of ionic species, particularly in aqueous environments that naturally contain other competing/inhibiting ions. Herein, we provide an overview of the technologies that have been developed, including the general theory, design, and benefits/challenges associated with ion-selective electrode technologies (carrier-doped membranes, carbon-based varieties, enzyme inhibition electrodes). Notable variations of these electrodes will be highlighted, and a brief overview of associated electrochemical techniques will be given.

## 1. Introduction

Ionic species are critical to chemical and biological processes all over the world. These charged species serve important functions in living organisms, such as the regulation of cardiovascular processes, and provide the foundation for a neural communication network in some animals. Charged species in the environment also facilitate plant growth and regulate the pH in aquatic ecosystems. Even though ions in low concentrations are often necessary for many biological processes, their presence in excessive concentrations can be toxic. These ions can become toxic in the body via a variety of routes. For example, heavy metals are known to inhibit important enzymes, generate free radicals, and have been associated with neurodegenerative diseases like Alzheimer’s and dementia [1,2,3]. Other ionic species have been known to act as transport inhibitors for important biomolecules and have also been studied as biomarkers and indicators of cancer [4,5]. Ions are ubiquitous in the environment in low concentrations. High concentrations of these species are often the result of pollution from mining and factories, as well as runoff from agricultural developments [1].

Toxic ionic species can enter the body by indirect means. A well-known example is the mercury content found in human food sources, especially fish and other aquatic species. In developing countries, drinking water can contain concentrations of heavy metals and other ions that are above the acceptable limits outlined by the World Health Organization (WHO) [6]. In developed countries, ions from old piping and industrial waste can make their way into food and water supplies. Therefore, a global interest in the development of a technology capable of furnishing precise measurements of analyte concentrations in aqueous solutions persists. Ideally, this technology would be robust enough for continuous remote monitoring in various aqueous compositions across a range of acidic or basic conditions.

While there are presently various sensor technologies that can be employed for the detection of ions in aqueous media, this review will focus specifically on technologies based on the electrochemical (EC) techniques. The operating principle of EC techniques is based on the interaction between the charged analyte species and a detector substrate via electron transfer. Typically, the substrate of an EC sensor is a conductive material connected to an instrument. Electron transfer between the substrate and the analyte species generates a current signal that can be used to determine the concentration of ions present in the water, given that precautions have been taken to ensure that the perceived activity of the analyte will be proportional to the actual concentration of the species of interest. Often, this interaction occurs at a characteristic potential for a given ion (especially in the case of heavy metal ion detection). This is convenient because it means that some EC techniques can utilize a single substrate to detect multiple ion-species.

EC techniques are a good candidate for aqueous ion-detection because they have been well-studied and harbor some favorable characteristics [7,8,9,10,11,12]. EC ion-detection is typically a low power technique and is relatively gentle on the environment as it generally involves low current and voltage values. Advancements in the miniaturization of the potentiostat, paired with its low-power requirement, mean that this technology can potentially be operated in remote places with limited resources [13]. EC methods for ion-detection are also desirable because the signal outputs of this technology, which are currents and voltages, interface well with modern electronics, computing, and signal processing techniques.

**Figure 1:**
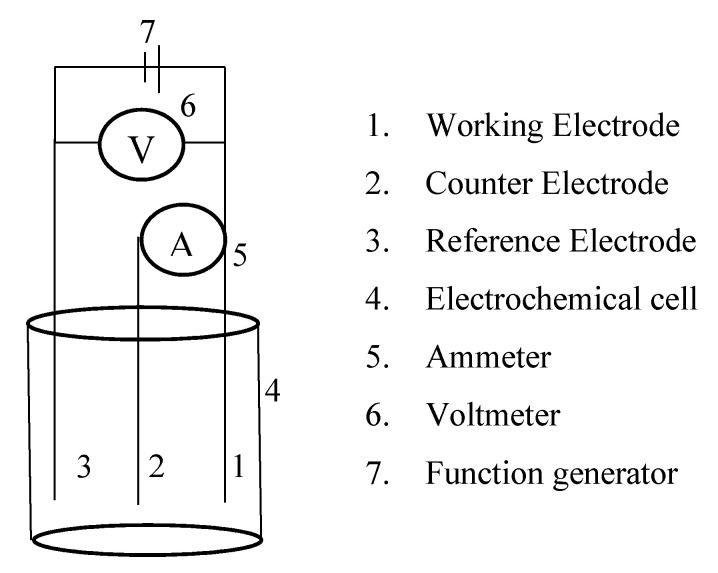
General electrochemical cell set-up. Typically, a potentiostat is used as the ammeter, voltmeter, and function generator.

EC methods rely on two components: the detector and the electroanalytical technique. In many cases, the detector is also called the “working electrode” and is paired with a reference electrode, and often a counter electrode as well (Figure 1). The materials selected for the reference and counter electrodes are often arbitrary but can be dependent on the composition of the solution if conditions are extreme. Typically, the counter electrode is an inert and conductive material like platinum. The reference electrode is usually a commercially available, high-stability electrode, such as the silver-silver chloride (Ag/AgCl) electrode. The working electrode contains an interface layer that interacts directly with the analyte of interest to produce an electrical response. A common practice is for an inert and conductive material to be coated or doped with another material capable of interacting with the analyte via electron transfer. Examples of this include various forms of carbon coated with nanoparticles, as well as other materials, such as polyvinyl chloride (PVC) doped with ionophores and “carrier” species capable of interacting with the analyte. This review will introduce some popular and novel designs of ion-selective electrodes, including carrier-doped membranes in addition to carbon-based, and enzyme-inhibition electrodes (Section 2).

The other fundamental component of EC technology is the electroanalytical technique that is employed. In general, there are three kinds of electroanalytical techniques: amperometry (measures current response), potentiometry (measures voltage response), and voltammetry (analyzes current-voltage relationship) [14]. Among them, voltammetry is the most developed and well-studied of electroanalytical techniques, but amperometry and potentiometry also have their advantages under certain situations. There are several exhaustive reviews and studies on these techniques [7,8,9,10], and this review will focus on the various forms of voltammetry (Section 3).

## 2. Electrode Designs and Considerations

### 2.1. Carrier-Doped Membrane Electrodes

**Figure 2:**
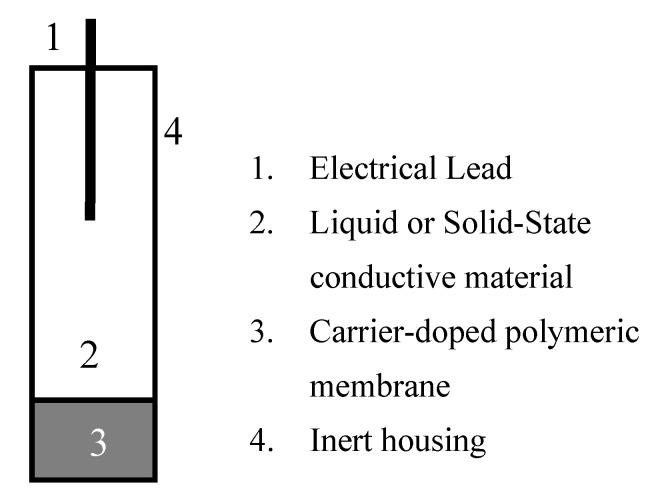
General design of a carrier-doped liquid-membrane electrode.

Carrier-doped membrane electrodes are one of the most well-studied ion-selective electrode (ISE) designs. The electrodes consist of a “liquid” polymeric membrane that has been doped with so-called “carrier” chemicals, which are typically molecules that exhibit electron-transfer activity when interacting with an analyte of interest. Since the carrier molecules are usually neutral, the membrane is typically doped with ionophores [15,16]—chemicals capable of attracting the analyte of interest—but will not inhibit it from interacting with the carrier. Finally, there is a conductive material inside of the membrane that carries the signal from the membrane to the measurement electronics. Traditionally, this material is a conductive liquid, however, solid conductive materials have become an increasingly popular choice due to the stable and versatile nature of solid-state and nano-materials [17,18,19,20,21,22,23,24,25,26,27]. Figure 2 provides a general representation of the structure and form of these electrodes. The different components that make up the carrier-doped membrane electrodes allow for customization of electrodes with tunability and versatility.

The major component of carrier-doped membrane electrodes is the membrane. Typical membrane matrices consist of polymer and plasticizer (also called membrane solvent) in a 1:2 (*w*/*w*) ratio. This high ratio of plasticizer to polymer helps to ensure that the constituents of the membrane (carriers, ionophores, etc.) are mobile and accessible enough to interact with the analyte [15,28]. The amount of plasticizer also influences the structural integrity of the electrode, so a balance between carrier mobility and structural rigidity needs to be established for an ideal electrode. The more plasticizer a membrane contains, the higher the chance that dopant material will leach into the sample solution/environment. As the electrode materials leach out, the performance of the electrode declines and the sample or environment is contaminated. However, if a membrane contains too little plasticizer, the decreased mobility of membrane constituents can manifest as an increase in specific membrane resistance. Generally, the issue of leaching can be solved by attaching lipophilic groups, such as long carbon chains, to the constituents of the polymer matrix [15,16,29]. Furthermore, the amount and type of plasticizer and polymer used can have an impact on the selectivity of the electrode. For example, if a membrane is doped with a non-selective carrier, the selectivity of the electrode is directly related to the difference in the standard free energy of that ion in the aqueous phase, versus the organic phase [15], which may affect the measurement accuracy in samples with multiple ionic species.

The polymer selected for fabrication of the membrane can have subtle effects on the performance of the electrode. Solubility of the polymer is the most important factor; the electrode must not be soluble in the sample or in the conductive liquid contained on the interior. Aside from solubility, one of the most influential factors for the polymer selected is the glass-transition temperature (*T_g_*). The *T_g_* of a polymer must be below room temperature, otherwise plasticizers will need to be included. This creates an interesting condition: membranes fabricated without plasticizers would totally avoid the issue of plasticizer leaching into the sample or environment. However, membranes fabricated with plasticizers have the additional benefit of tunability via control of plasticizer type and amount. In addition, many polymers can be electrically insulating. Thus, the presence of impurities and ionophores, as well as the ratio of polymer to plasticizer composition, can mitigate the insulating effects of the membrane matrix. Arada-Pérez et al. recently determined that there is no correlation between the selectivity of an electrode and the type of plasticizer, however, they did establish a positive inverse correlation between plasticizer viscosity and electrode longevity (lower viscosity plasticizers resulted in longer electrode lifetimes) [28].

The most critical consideration in the fabrication of carrier-doped membrane electrodes is the selection of the carrier itself. This is because the carrier serves as the primary means of ion-selectivity in the matrix. Generally, carriers are molecules that specifically interact with the analyte in such a way that leads to electron transfer from the membrane to the interior conductive liquid. Although selectivity for a particular analyte can be attributed to various chemical and physical phenomena, modern understanding of Host–Guest interactions supports the size-exclusion, charge, and hydrophobicity as the most influential characteristics contributing to selectivity [30]. Neutral carriers are typically favored over charged carriers for multiple reasons. The most significant reason for selecting a neutral carrier is that these carriers are less likely to attract a competing ion than charged carriers. In addition, neutral carriers are less likely to be inhibited by the analyte and thereby more likely to exhibit weak and reversible interactions favored for sensing applications [15]. Carriers with lipophilic characteristics are also favorable because they stay in the polymer matrix better, eliminating some issues associated with leaching. In light of these considerations, organic ligands make excellent candidates as carriers for electrodes focused on sensing metal ions [15,20,21,29]. In fact, their tendency to have specific interactions and weak coordinate bonds between the ligands and metal centers mean that the interactions are more likely to be reversible. Furthermore, the carbon framework in ligands can help contribute to the lipophilicity of the carrier, and therefore the robustness of the electrode.

If a neutral carrier is used, then the membrane must be doped with an ionophore to help attract analyte molecules to the carrier. Essentially, an ionophore is an ion included in the membrane matrix that is opposite in charge to the analyte of interest. The choice of ionophore can have a major impact on the performance of an electrode, as well as the applications that electrode might be suitable for [16]. Typical ionophores are salts where the ionophore is lipophilic or has a lipophilic group attached. The lipophilicity of the ionophore is a critical factor in preventing leaching from the electrode into the sample or environment. Another important factor is the nature in which the ionophore interacts with the analyte. Recent studies by Bakker et al. have shown that the low detection limits of carrier-based ion selective electrodes (ISEs) are strongly dependent on the nature of the ionophore; more stable ion-ionophore complexes are associated with lower detection limits [29].

In general, carrier-doped membrane based electrodes are desirable for sensing applications both in vivo and in vitro. These electrodes have been studied for decades now and the general design easily lends itself to modification and tunability. In addition, the bulk of materials required to fabricate these electrodes are widely available in industry. The application of doped-membrane ISEs are practically limitless, as shown by Diamond et al. who recently developed a multi-electrode potentiometric sensor capable of speciating Pb^2+^ ions in an aqueous solution while simultaneously monitoring the pH of the solution [22]. The largest drawback of these electrodes is due to their construction and the way they operate: they are prone to leaching and are generally not robust enough for extended sensing in real-world applications. In addition to leaching, the membranes can also be prone to uptake and retention of the analyte, which results in inaccurate measurements.

Table 1 lists some common carrier-doped electrodes and their performance and specifications, such as stability, response time, limit of detection (LOD), etc. This table is intended to provide a range of data on the possible materials and capabilities of this technology.

### 2.2. Carbon-Based Electrodes

Carbon-based electrodes are a popular choice for many electrochemical measurements. This is because carbon is generally chemically inert under most electrochemical conditions, is conductive and non-toxic, and can also withstand a wide range of potentials without perceivable damage to the electrode. It should also be noted that carbon electrodes show electrocatalytic activity in biomolecular redox reactions, which allows carbon electrodes to be employed effectively in the detection of biomolecules [35]. In general, any ion-specific selectivity observed in carbon-based electrodes is significantly dependent on the material they are modified or doped with, rather than the structure/composition of the carbonaceous material.

The allotropes of carbon provide an advantage in the sense that carbon is available in several different structures and forms, meaning that there will be several available routes for fine-tuning and modifying carbon-based electrode materials. There are more economic forms of carbon, such as graphite, that can be utilized for common applications that do not require a more ordered form of carbon. However, it is uncommon to see these forms of carbon used, as they are often laden with impurities. Some higher quality forms of carbon, including diamond and glassy/vitreous carbon, are more appropriate for sensing applications.

#### 2.2.1. Diamond Electrodes

Diamond is an sp^3^ hybridized allotrope of carbon that is renowned for its hardness and general robustness. The tetrahedral bonding nature of diamond results in a low conductivity material. As a consequence, only diamond that has been doped with an impurity like boron or nitrogen makes a good candidate for consideration as an electrode material. Boron-doped nanocrystalline diamond is the most popular form of diamond utilized for electrode materials [35]. This material is significantly different from bulk diamond, as the randomly-oriented crystals are both n-type and p-type doped, introducing sp^2^ hybridized structures that significantly increase the electrical conductivity.

#### 2.2.2. Glassy-Carbon Electrodes

Glassy carbon is an sp^2^ hybridized form of carbon that shares similar characteristics to graphite, but shows increased hardness and thermal resistance, as well as good conductivity, owing to the electronic nature of sp^2^ carbon. As a result, these forms of carbon are typically modified at the surface. Glassy carbon is considered to be a variant of graphite and is generated from treating polymeric materials with significant heat under an inert atmosphere.

#### 2.2.3. Carbon Paste Electrodes

For special applications where the entire sensing element needs to be of similar composition throughout, or the electrode surface area needs to be significantly larger, carbon-paste electrodes, carbon nano-tubes (CNTs), and multi-walled carbon nano-tubes (MWCNTs) can offer particulate-type alternatives to diamond and glassy carbon [35,36,37,38]. A significant advantage of these materials, aside from their increased surface area and improved ability to mix with dopants/modifiers, is that these materials are moldable/formable and can be made into any shape/size easily without significantly impacting the performance of the electrode.

Carbon paste electrodes (CPEs) are traditionally formed by the mixture of graphite powder with paraffin oils or silicone fluids that has been packed into a housing made of glass or other inert and robust materials [36]. These electrodes have the significant advantage of being fabricated from economic and industrially available materials. The nature of their fabrication also makes these electrodes good candidates for modification, as the modifier can be mixed in and evenly dispersed throughout the paste. Modern CPEs are also constructed from other carbonaceous materials, like CNTs, fullerenes, diamond, and even powders of glassy carbon, further adding to their versatile and tunable nature [36].

#### 2.2.4. Carbon Nanotubes Electrodes

Carbon nanotubes (CNTs) share many of the beneficial characteristics of other popular carbon electrode materials: they are generally chemically inert, conductive, and have good thermal resistivity and stability. In addition to these characteristics, CNTs have a significantly higher surface area than other carbon materials, and are functional and practical while boasting a small size, making them ideal candidates for biosensors [37]. CNTs employed in electrodes have also been known to reduce overpotential, increase sensitivity, and even show enhanced reversibility [38]. CNTs are best visualized as a graphene-like sheet of sp^2^ carbon that has been rolled into a tube. Single walled CNTs are simply a single sheet that has been rolled, with diameters ranging from 0.4 nm to 2 nm. Multi-Walled CNTs (MWCNTs) consist of concentric tubes around a singular axis; these concentric tubes are typically spaced 0.34 nm apart and the diameter of the total structure can range from 2 nm to 100 nm. Single walled CNTs exhibit metallic and semiconductor characteristics, while MWCNTs have the desirable property of being exclusively metallic [37]. As a lone material, CNTs do not make for a robust macrostructure. As a result, they are often used as a modification or component in an electrode rather than being utilized in a standalone fashion [17,31,33,38,39,40,41,42]. The ends of CNTs can be capped with fullerene-like structures or can be modified with functional groups, which opens opportunities for selectivity and further modification [35,37]. CNTs are generally synthesized through catalytic chemical vapor deposition (CCVD), arc discharge synthesis, or laser ablation/vaporization [38].

CNTs have been incorporated into electrode designs by inclusion in a binder, drop-coating, forming a paste similar to carbon paste, and formation of composite materials that include CNTs [17,31,33,38,39]. The tubes are typically randomly aligned. However, there are many techniques for producing aligned CNTs, including metal-assisted self-assembly mechanisms, growth from a substrate, extrusion while in a polymeric binder, and exploitation of the boule that forms during arc discharge synthesis of MWCNTs. Aligned bundles of CNTs boast more desirable electrical characteristics than other forms of CNTs. This is because in the aligned CNTs, the electrochemical interactions are dominated by the ends of the tubes rather than the walls of the tubes [35]. This is ideal because the conduction of electrons occurs from end to end. In addition, these bundles harbor a different surface chemistry compared to scattered tubes, since the ends of CNTs are hydrophilic while the walls are hydrophobic. This further adds to the versatility of these materials for application in electrochemistry by allowing for modification and functionalization by materials that are hydrophilic, which are of particular interest due to the intimate relationship between charge and hydrophilicity [37].

Some common carbon-based electrodes are listed in Table 2. Their sensing performances, such as the detection limits, pH range, sensitivity, response time, etc., are also included for comparison purposes.

### 2.3. Enzyme-Based Electrodes

**Figure 3:**
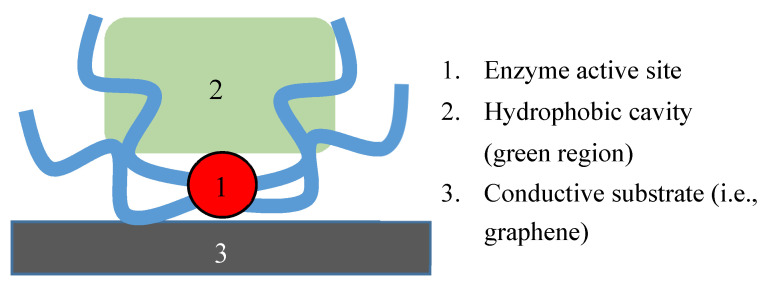
General enzyme features relevant to sensing technology.

Electrodes fabricated using enzymes can exhibit excellent selectivity and reversibility, owing to the naturally selective and catalytic nature of enzymes. In almost all modern cases, the enzyme is immobilized on the surface or inside a carbon-based material or a conductive polymer and then that material is layered onto a more traditional electrode material. Graphene has become an increasingly popular material for use with enzymes in the construction of electrochemical sensors [50]. This is due to the significant electron-transport abilities of graphene, which enhances the electron transfer from the enzyme to the electrode material. The enhanced electron-transport abilities of graphene and CNTs are desired, and in most cases required, because most enzymatic active sites are located in a hydrophobic cavity of the molecule that is insulated by the protein layers (Figure 3). Therefore, it is critical that any redox activity results in electron transfer to the electrode. Gold and other metals with high electron transport characteristics can serve as alternative substrates to graphene and CNTs. More bio-compatible and economic materials, such as pectin [51], have been explored for the immobilization and application of enzymes to sensing technology. Redox enzymes (enzymes that undergo electron-transfer through reduction–oxidation reactions) are the best candidate for this kind of sensor, as the electron transfer that happens at the catalytic center of the enzyme can be conducted directly into a graphene/CNT substrate, and then into a traditional electrode [50,52].

While enzymatic sensors have been employed successfully, they remain one of the less-popular options for most sensing applications for a variety of reasons. One of the most significant factors impacting the use and research of enzyme-inhibition electrodes is that isolated enzymes can be quite expensive, and characterization and storage of enzyme-based electrodes often requires special considerations such as refrigeration [51] when compared to traditional electrode materials. Another factor significantly impacting the application of enzymatic sensors is that biomolecules tend to be more sensitive to their chemical environment than traditional electrode materials, and as a result are confined within a smaller window of pH and will be less tolerable to high potential [53]. In addition to this, many enzymes can be inhibited by a particular ion [1,3]. In some cases, this interaction may be favorable for sensing, if it can be reversed. If enzymes are irreversibly inhibited, however, the electrode is rendered inoperable unless restored by some other chemical means.

In-spite of the drawbacks listed above, enzymatic sensors can be powerful tools for special applications where the conditions are favorable for the device. A notable feature of these devices is that the selectivity is determined almost exclusively by the nature of the enzyme. This is significant because it means that these devices will be much more difficult to tune in terms of selectivity, i.e., trying to detect a similar ion by making small changes in design will prove more difficult. In some ways, this is a serious advantage: multiple enzymes can be included in a single electrode design for monitoring of multiple ions, typically without interference or affecting the performance of the other enzymes [52]. Multiple-ion-selectivity is a capability that becomes significantly more complex outside of an enzymatic approach. In addition, the fundamental approach to designing and fabricating these devices for the sensing of specific ions is uniquely simple in nature compared to the other electrode designs, in the sense that typically no further modifications are required for selectivity. This simplicity makes enzymatic sensors an attractive option for primary studies where selectivity is desired and a common or commercial alternative sensor has not been developed [53,54].

Table 3 lists some common enzyme-based electrodes and their sensing performances, such as the detection limits, pH range, sensitivity, response time, etc., are also included.

## 3. Electrochemical Techniques

Voltammetry remains one of the most commonly-used and powerful techniques available in the application of electrochemistry. Voltammetric techniques focus on the relationship observed when a current response is measured as a function of the potential. In general, electroanalytical voltammetry employs a three-electrode cell design with a potentiostat, in which the potential is applied between a working electrode and reference electrode [14]. The working electrode is the electrode at which the electrochemical reaction of interest is occurring and the reference electrode is a material with constant potential. A counter electrode is employed near the working electrode to serve as an electron reservoir and measure the current that is generated by the redox activity occurring at the working electrode. Typically, an electrolyte is included in the solution to enhance conductivity between the electrodes [14].

Amperometry is another popular electrochemical method, in which the potential is held at a set value optimized for detecting a current response from the analyte. In this case, the magnitude of the current signal and the way that it changes are used to furnish information about the analyte. In general, this technique can be viewed as a simple form of voltammetry where the potential is constant or pulsed to a constant magnitude. Hence, this review does not provide detailed discussion on amperometry.

There is plethora of resources that discuss the chemical and kinetic challenges presented by voltammetry [7,8,9,10,14,69]. This section will present the general performance and application to ion-sensing of the different potential wave forms commonly used in electroanalytical techniques. In general, the applied potential is often related to the identity of the ion being analyzed, while the current response can be indicative of the amount of ion present in the sample.

### 3.1. Sweep Voltammetry

**Figure 4:**
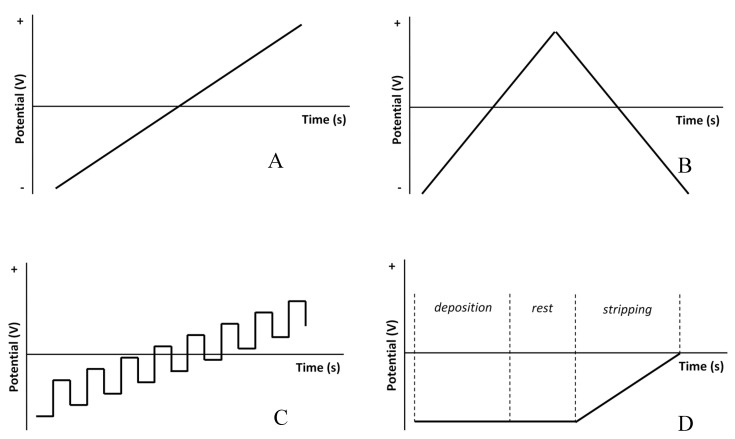
(**A**) Potential vs. time signal for linear sweep; (**B**) Potential vs. time signal for cyclic voltammetry; (**C**) Potential vs. time signal for square wave voltammetry; (**D**) Potential vs. time signal for stripping voltammetry. Anodic stripping is shown in this case.

One of the most widely-used forms of voltammetry, sweep voltammetry involves measuring the current as a function of the potential, which is varied with time at a constant rate. In sweep voltammetry, the data that is of interest is the peak current and the peak potential. The most basic form of sweep voltammetry is linear sweep voltammetry (LSV), in which the potential is varied at a constant rate in a single direction and the current response is measured [10,11], as illustrated by Figure 4A. LSV introduces an important nuance that applies to all electroanalytical voltammetric techniques that utilize a dynamic potential. Since the electrochemical cell behaves as a small capacitor, any time the potential is changed, a charging current flows. If the potential is stationary, this current disappears. However, as the potential is continuously being changed in LSV (as well as other techniques), there is always a charging current that flows. Therefore, any response current measured in a dynamic potential setting should be corrected for this phenomenon by subtracting the charging current from the peak current signal. There are simple, standardized techniques for determining the charging current [14].

Cyclic voltammetry (CV) is almost identical to LSV, except that the experiment is performed in reverse once a particular time or potential is reached, as shown by Figure 4B. For instance, if a cathodic scan is done, it will be followed by an anodic scan, and vice-versa. In some cases, the scan-rate in the reverse direction is altered from the scan-rate in the forward direction, however this is not a common practice [12,14]. A simple reason for this is that one of the advantages of CV is the ability to study the electrochemical cell’s behavior in reverse operation, and the comparison of behavior in the forward and reverse scans at two different scan rates is difficult due to the stronger charging current generated by the faster scan-rate. This means that correcting for the charging current will be different for forward and reverse scans unless the same scan-rate is shared. Another unique feature of CV is that the effect of consecutive cycling on the response behavior of the cell can be studied by superimposing the CVs of multiple cycles taken in succession. In the development of ion-sensing technology, this could give information about the robustness and reliability of a sensing apparatus.

In CV, the peak current and peak potential of both cathodic and anodic sweeps can be compared in order to furnish additional information, such as reversibility and electron transfer kinetics, from the electrochemical cell [12]. Deviation of the ratio of anodic to cathodic peak currents across different cycles is associated with homogenous kinetic phenomenon and other electrode process issues. The separation of potential peaks can be an indicator for how well a given electrochemical process fits Nernstian behavior. In general, the difference in potential for anodic and cathodic peak potential is expected to be close to 2.3 *RT*/*nF* where *R* is the universal gas constant, *T* is temperature in Kelvin, *n* is an integer related to the number of electrons transferred for the electrochemical reaction, and *F* is Faraday’s constant [14].

### 3.2. Square Wave Voltammetry

Square wave voltammetry (SWV) is best described as a form of voltammetry in which the potential wave form is the result of the super position of a square potential wave form and a staircase potential wave form (Figure 4C). The square wave potential allows for the double layer charging current to be treated as negligible, given that sufficient time has passed between the initiation of the pulse and time of the measurement. By reducing the effect of the charging current on the measurement, more minute and precise changes in current can be detected. This is a very desirable effect, especially in the realm of detecting trace amounts of ionic species. The success of this concept of SWV revolves around the fact that charging currents decay at a significantly faster rate than the Faradaic currents associated with electrochemical activity. The amount of time that passes between pulse initiation and current measurement is determined by minimizing the effect of the charging current in both anodic and cathodic current responses [8]. The staircase potential allows this technique to be swept across a range of potentials without losing the advantages gained from the square-shaped pulse [7].

Another unique feature of this technique is that it results in a current response peak that is symmetrical in shape, rather than the asymmetrical peaks that are associated with LSV and CV. This is achieved because the final waveform for the current signal is the differential sum of the cathodic and anodic current response peaks, which occur in consecutive half-cycles. The combination of symmetrical peak output and compensation for the charging current, caused by the double layer capacitance, have made this a popular technique for electroanalytical applications that require increased performance compared to sweep voltammetry methods [8].

### 3.3. Differential Pulse Voltammetry

Differential pulse voltammetry (DPV) was the predecessor to SWV and operates under the exact same principles, except that in SWV, an equal amount of time is spent at the ramped baseline potential and the pulse potential. In DPV, the super-imposed pulse potential is typically shorter than the various steps of the baseline/staircase potential.

### 3.4. Stripping Voltammetry

Stripping voltammetry is an electroanalytical technique that was developed to improve the sensitivity and accuracy of measurements, particularly in the case of analytes that exist in trace amounts. While stripping voltammetry can be performed with a cathodic or anodic potential, the most popular form of stripping analysis is anodic stripping voltammetry (ASV). This is due to this technique’s success in the detection of metal ions, which are generally cationic. The distinguishing feature of this technique is a pre-electrolysis step in which the analyte of interest is pre-concentrated onto the working electrode’s surface. In the case of ASV, this concentration step is done at a cathodic potential that is within the electrochemical window of the solution being investigated, but also sufficiently past the reduction potential for the analyte of interest. Depending on the electrode employed, there may also be a rest-period after deposition to allow the analyte to equilibrate in the electrode media. Figure 4D illustrates a time-dependent potentiometric signal for electrolysis prior to stripping. It is critical that the solution/electrode interface maintains constant during this step for accurate analysis. This is typically achieved by either stirring the solution at a constant and repeatable rate, or by the employment of a rotating-disc electrode. After this deposition, the analyte of interest is then “stripped” via LSV or DPV with a potential opposite in nature to the pre-electrolysis step [14].

Although thorough electrolysis of the entire solution is not necessary, this technique relies heavily on calibration, and therefore is not usually the technique of choice for the primary investigation of electrochemical systems. In addition to this, there is also an issue of residual analyte on the surface of the electrode after the stripping analysis. Therefore, it is good practice to include a “cleaning” step, in which the potential is held past the oxidizing potential of the analyte for an extended period of time. In spite of these extra precautions, however, the issue of irreversible deposition of analyte onto electrode surfaces has been an unresolved issue since the conception of this technique [69,70,71,72].

Stripping voltammetry has also been utilized successfully in the simultaneous determination of multiple analyte species. This tends to work well in the detection of ionic species with redox potentials that are well-separated, but still within the working electrochemical window of the solution. The only significant drawback introduced by this technique is that pre-concentration and analysis steps that would normally be optimized for a single analyte must now be optimized for multiple analytes, which means that the potential performance of the technique is diminished slightly for every new analyte that needs to be detected.

## 4. Conclusions

Three selective electrode designs are presented in this review as viable modern tools for the investigation of chemical species. In particular, carrier-doped electrodes feature a highly-tunable design that makes them an ideal choice for most research applications. The leaching effect of these electrodes, however, can be a significant drawback in environmental and biological applications [15,16,29] and limiting their long term use. Carbon-based electrodes are ideal for most sensing applications since carbon comes in many forms, is conductive, inert, and relatively robust. The inert nature of carbon materials renders them a favorable choice for the fabrication of in-vivo sensors and analyses that demand a material with good electrochemical stability [35,36]. Enzyme-based sensors are not as common as carrier-doped or carbon-based electrodes, likely due to the cost and storage requirements of enzymatic technology [60,63,66]. However, they fill an important niche in selective sensing. The selective and biochemical nature of enzymes makes them a desirable choice for sensitive biological systems and medicinal applications [41,52,53,54,73].

The electrodes discussed in this review and catalogued in the tables have been successfully applied in both environmental and medical applications. Selective sensors undoubtedly have a major role to play in the medical field: sensors have been developed to screen for cancer [4,5] and bodily fluids for important biomolecules [51,66,74]. The sensors also have major applications in the field of environmental monitoring. Suprun et al. developed a bi-enzyme sensor capable of monitoring several different anilines and multiple pesticides [57]. Yu, et al. recently fabricated a carrier-doped electrode capable of monitoring Pb^2+^ concentrations in real-time [26].

In spite of the benefits associated with these techniques, there are currently some limitations associated with the EC technology for environmental sensing. One of the biggest challenges facing EC technology is that the concentration of analyte species that can interface with the electrode should remain relatively constant during the analysis process. This can prove difficult in natural waterways where turbulence and stagnation can both be issues, or in cases where the electrochemical reaction being exploited to gather data is not totally reversible. Rotating disc electrodes are an example of simple and yet effective EC technology that has been developed to furnish a more “constant” environment for ion-detection. However, the issue pertaining to the reversibility of the interaction between analyte and substrate is more complex and is often dependent on several physical and chemical characteristics associated with the specific electrode’s design. Another major challenge associated with EC technology for ion-detection is the sensitivity of the technique to impurities in the sample. Indeed, competing ions, natural debris, and even dissolved gases can have a significant impact on EC measurement.

Several common voltammetry techniques that are typically paired with the selective electrodes have also been discussed. In-general, techniques like square wave voltammetry tend to furnish the lowest limits of detection (LOD) and with faster response times [7,8,9]. Cyclic voltammetry is a special form of linear sweep voltammetry that can give information about the reverse electrochemical reaction and the long-term behavior of an electrochemical cell [10,14]. Stripping voltammetry, on the other hand, can be a good choice in situations where a lower LOD is desired than with other methods. However, this method suffers from heavy dependence on calibration and repeatable conditions, as well as accumulation of the analyte at the electrode surface [69,70,71,72].

The future of environmental and medical sensing will most certainly be ushered-in by the technologies stemming from modified carbon and enzyme-based electrodes, owing to the high bio-compatibility and high-selectivity furnished by these materials respectively. These technologies will likely be paired with square-wave and cyclic voltammetry when applied in the field, as these techniques tend to furnish the most information while retaining high selectivity and low detection limits [7,12]. Additional studies on the amplification and interpretation of electrical signals should be undertaken in order to utilize the full potential of electrochemical techniques [75]. Further work in understanding selectivity, enzymes, and the development of bio-mimic molecules capable of replicating the selectivity of enzymes will be critical in the development of future sensing technology [13,54,58,76].

## Figures and Tables

**Table 1:** Carrier-doped electrodes and their performance.

Electrode Material	Analyte	LOD (M)	Concentration Range (M)	pH Range	Nernstian Slope (mV/decade)	Longevity	Response Time (s)
[17] CNT-Silver Borate epoxy composite	BO_3_^3-^ (borate)	2 × 10^−5^	10^−1^–10^−4^	4–8	34	3 months	14
[19] screen printed carbon-contact with Chitosan Prussian Blue nanocomposite and doped PVC membrane	Na^+^		1–10^−4^		52.4	20 h	
[20] doped PVC with graphite-epoxy contact	Cl^-^		10^−2^–10^−6^	2–6	63.43 ± 0.85	15 days	25
[21] doped PVC with graphite-epoxy contact	Cu^2+^	6.15 × 10^−7^	10^−2^–10^−7^	8–10	29.35 ± 0.6	20 days	20
[22] doped PVC with PEDOT/gold contact, multi-electrode device	Pb^2+^	~10^−8^		2–9	59.8 ± 0.1	3 months	~120
[23] doped PVC with polypyrrole/carbon paste contact	Hg^2+^	6 × 10^−10^	10^−2^–10^−9^	3–4	23.7 ± 1.4		60
[24] doped PVC on platinum contact	Y^+3^	2.15 × 10^−9^	10^−1^–10^−8^	2–6	18.89 ± 0.43		~15
[25] doped PVC on DH-6T/gold contact	Pb^2+^	3.16 × 10^−7^	10^−2^–10^−7^		28.1 ± 0.4		
[28] doped PVC on graphite-epoxy contact	NO_3_^-^ (nitrate)	1.95 × 10^−5^			-59.9 ± 0.9	>6 months	
[26] doped PVC on F8BT/gold contact	Pb^2+^	3 × 10^−8^	10^−3^–10^−8^		30.1		~10–15
[27] spray-coated doped PVC on spray-coated CNTs	K^+^	3.16 × 10^−7^	0.1–10^−9^		59.8 ± 0.4	>2 weeks	~20
[27] spray-coated doped PVC on spray-coated CNTs	H^+^		10^−1^–10^−5^		53.7 ± 1.1		~20
[27] spray-coated doped PVC on spray-coated CNTs	Cl^-^		10^−1^–10^−5^		-56.3 ± 1.3		~20
[31] doped PVC on CNT modified GC electrode	Pb^2+^		10^−3^–10^−8^		29.0 ± 0.8		~5–20
[32] doped polypyrrole on graphene oxide modified GC electrode	Pb^2+^ Cd^2+^		7.2 × 10^−7^–4 × 10^−8^	4.5		Several weeks	
[33] doped poly(MMA*-co-*BA) on MWCNT modified Au disc electrode	Pb^2+^	10^−10^	1.5 × 10^−3^–2.0 × 10^−10^		29.1 ± 0.5		
[34] doped PVC on graphite rod	Eu^3+^		1 × 10^−1^–5.7 × 10^−8^	2.7–9.0	19.5 ± 0.2		10

Abbreviations: LOD, limit of detection; PVC, polyvinyl chloride; CNT, carbon nanotubes; PEDOT, poly(3,4-ethylenedioxythiophene); GC, glassy carbon; F8BT, poly(9,9-dioctylfluorene-alt-benxothiadiazole); MMA, methylmethacrylate; BA, butyl acrylate; DH-6T, 5,5’’’’’-dihexyl-2,2’:5’,2’’:5’’,2’’’:5’’’,2’’’’:5’’’’,2’’’’’-sexithiophene; MWCNT, multi-walled carbon nanotubes.

**Table 2:** Carbon-based electrodes and their performance.

Electrode Material	Analyte	LOD (M)	Concentration Range (M)	pH Range	Longevity	RT (s)
[43] Au nanoplates on reduced graphene oxide modified GC electrode	ascorbic acid dopamine * uric acid **	5.1 × 10^−5^ 1.4 × 10^−6^ * 1.8 × 10^−6^ **	1.5 × 10^−3^–2.4 × 10^−4^ 41. × 10^−5^–6.8 × 10^−6^ * 5.3 × 10^−5^–8.8 × 10^−6^ **		7 days	
[44] Fe_2_O_3_ and reduced graphene oxide composite modified GC electrode	NO_2_^−^ (nitrite)	1.5 × 10^−8^	7.8 × 10^−4^–5.0 × 10^−8^		10 days	
[45] graphene-modified GC electrode	Hydroquinone * Catechol **	1.5 × 10^−8^ * 1.0 × 10^−8^ **	5 × 10^−5^–1 × 10^−6^	~4.5–6.2	3 weeks	
[5] gold nanorods on graphene-oxide modified GC electrode	miRNA-155	6 × 10^−16^	8 × 10^−12^–2 × 10^−15^			
[46] molecularly imprinted polypyrrole on graphene quantum dot modified GC electrode	Bisphenol A	4 × 10^−8^	5 × 10^−5^–1 × 10^−7^		15 days	
[47] imprinted zeolite in carbon paste	creatinine	7.9 × 10^−8^	10^−5^–10^−7^	7	7 weeks	<50
[48] Au nanoparticles on flower-graphene modified GC electrode	NO_2_^-^ (nitrite)	1 × 10^−8^	2.04 × 10^−2^–1.25 × 10^−6^			
[39] ferrocenedicarboxylic acid with MWCNTs in carbon paste	6-thioguanine Folic acid *	8.5 × 10^−9^ 1.1 × 10^−6^ *	1 × 10^−4^–1 × 10^−8^ 1.52 × 10^−4^–4.6 × 10^−6^ *	9	20 days	
[28] ionophore-doped MWCNT-ionic liquid paste	Hg^2+^	2.5 × 10^−9^	1.0 × 10^−4^–5.0 × 10^−9^	2.0–4.3	>55 days	~5
[49] reduced graphene oxide screen-printed on PVC	ascorbic acid dopamine * uric acid **	9.5 × 10^−7^ 1.2 × 10^−7^ * 2.0 × 10^−7^ **	4.5 × 10^−3^–4.0 × 10^−6^ 2.0 × 10^−3^–5 × 10^−7^ * 2.5 × 10^−3^–8 × 10^−7^ **			~10–15
[42] palladium, carbon nanofiber nanocomposites on PVC	ascorbic acid dopamine * uric acid **	1.5 × 10^−5^ 2 × 10^−7^ * 7 × 10^−7^ **	4 × 10^−3^–5 × 10^−5^ 1.6 × 10^−4^–5 × 10^−7^ * 2 × 10^−4^–2 × 10^−6^ **			

Asterisks in this table are used to relate multiple lines of data for different analytes in a single work. Values marked with (*) or (**) indicate that the value corresponds to the analyte with the same symbol. Abbreviations: RT, response time.

**Table 3:** Enzyme-based electrodes and their performance.

Electrode Material	Analyte	LOD (M)	Sensitivity (μA/mM)	pH Range	Longevity	RT (s)
[55] xanthine oxidase in carbon paste (1,4-benzoquinone modification)	Xanthine	1 × 10^−7^	6.91	7.0–8.5	14 days	100
[55] xanthine oxidase in carbon paste (PVF modification)	Xanthine	1 × 10^−7^	4.61	7.0–8.5	7 days	50
[56] formaldehyde dehydrogenase on graphite gauze	formaldehyde (gas-phase)	0.03 ppm	2 μA/ppm	6.5–8.5		300
[57] cholinesterase and peroxidase immobilized in polytyramine on epoxy-carbon film	aniline hydroquinone coumaphos chlorpyrifos-methyl	3 × 10^−8^– 3 × 10^−4^	1.3-1126			
[58] acetylcholine esterase biomimic immobilized in acrylamide and BIS on carbon	acetylcholine	4 × 10^−3^				
[59] tyrosinase immobilized on carbon nanotube paste modified with cobalt phthalocyanine	Catechol * Catechin **	1.66 × 10^−6^ * 6.32 × 10^−6^ **	64.0 *	7.0	<1 month	
[51] enzymes immobilized in pectin on screen-printed graphite	Glucose Sucrose D-glucose Ascorbic Acid				25 weeks	
[60] glucose oxidase immobilized in MPC-*co*-PMD on dissolved oxygen electrode	glucose	1.95 × 10^−5^	0.992		<72 hr at RT 14 days at 4 °C	51.8
[61] xanthine oxidase in Immunodyne® ABC membrane in Teflon on oxygen electrode	hypoxanthine				2 months at 40 °C	
[62] alcohol dehydrogenase and coenzyme immobilized in polyaniline on gold-coated polycarbonate	ethanol	0.092		7.0		~15 min
[63] glucose oxidase with C-60 fullerene immobilized on silica and PZ quartz	glucose	3.9 × 10^−5^			>3 months	
[64] glucose oxidase and horseradish peroxidase immobilized in concanavalin A on GC electrode	glucose		9.5–15.0	~7	<1 month @ 4 °C	~20
[65] glucose oxidase immobilized in cellulose on GC electrode	glucose	1 × 10^−5^	~0.125		6 months @ 4 °C	~10
[66] ß-galactosidase and glucose oxidase electrodeposited on Pt	lactose	<1.4 × 10^−2^	0.111 ± 0.002	4.9		~8
[67] urease with PAMAM and CNTs on FETs	urea	<1 × 10^−4^				
[68] lactate oxidase with TTF and Nafion on GC electrode	lactate	<5 × 10^−4^		6.5–8.0	~2 months	40

Values marked with (*) or (**) indicate that the value corresponds to the analyte with the same symbol. Abbreviations: TTF, tetrathiafulvalene; PAMAM poly(amidoamine); FET, field-effect transistor; MPC, 2-methacryloyloxyethyl phosphorylcholine; PMD, dodecylmethacrylate; PZ, piezoelectric; PVF, poly(vinylferrocene); BIS, N’N-methylene-bis-acrylamide.

## References

[1] Florea A.M., Busselberg D. (2006). Occurrence, use and potential toxic effects of metals and metal compounds. BioMetals.

[2] Camargo J.A., Alonso A. (2006). Ecological and toxicological effects of inorganic nitrogen pollution in aquatic ecosystems—A global assessment. Environ. Int..

[3] Gumpu M.B., Sethuraman S., Krishnan U.M., Rayappan J.B.B. (2015). A review on detection of heavy metal ions in water—An electrochemical approach. Sens. Actuators B Chem..

[4] Zhu Y., Wang H., Want L., Zhu J., Jiang W. (2016). Cascade Signal Amplification Based on Copper Nanoparticle-Reported Rolling Circle Amplification for Ultrasensitive Electrochemical Detection of the Prostate Cancer Biomarker. ACS Appl. Mater. Interfaces.

[5] Azimzadeh M., Rahaie M., Nasirizadeh N., Ashtari K., Naderi-Manesh H. (2016). An electrochemical nanobiosensor for plasma miRNA-155, based on graphene oxide and gold nanorod, for early detection of breast cancer. Biosens. Bioelectron..

[6] World Health Organization Guidelines for Drinking-Water Quality, Fourth Edition. https://www.who.int/water_sanitation_health/publications/2011/9789241548151_ch12.pdf.

[7] Osteryoung J.G., Osteryoung R.A. (1985). Square Wave Voltammetry. Anal. Chem..

[8] Ramaley L., Krause M.S. (1969). Theory of Square Wave Voltammetry. Anal. Chem..

[9] O’Dea J.J., Osteryoung J., Osteryoung R.A. (1981). Theory of Square Wave Voltammetry for Kinetic Systems. Anal. Chem..

[10] Laviron E. (1979). The use of linear potential sweep voltammetry and of ac voltammetry for the study of the surface electrochemical reaction of strongly adsorbed systems and of redox modified electrodes. J. Electroanal. Chem..

[11] Nadjo L., Saveant J.M. (1973). Linear sweep voltammetry: Kinetic control by charge transfer and/or secondary chemical reactions. Electroanal. Chem. Interfacial Electrochem..

[12] Nicholson R.S. (1965). Theory and Application of Cyclic Voltammetry for Measurement of Electrode Kinetics. Anal. Chem..

[13] Hoilett O.S., Walker J.F., Balash B.M., Jaras N.J., Boppana S., Linnes J.C. (2020). KickStat: A Coin-Sized Potentiostat for High-Resolution Electrochemical Analysis. Sensors.

[14] Bard A.J., Faulkner L.R. (2000). Electrochemical Methods.

[15] Bakker E., Buhlmann P., Pretsch E. (1997). Carrier-Based Ion-Selective electrodes and Bulk Optodes. 1. General Characteristics. Chem. Rev..

[16] Buhlmann P., Pretsch E., Bakker E. (1998). Carrier-Based Ion-Selective Electrodes and Bulk Optodes. 2. Ionophores for Potentiometric and Optical Sensors. Chem. Rev..

[17] Bahar D.U., Topcu C., Ozcimen D., Isildak I. (2020). A Novel Borate Ion Selective Electrode Based On Carbon Nanotube-Silver Borate. Int. J. Electrochem. Sci..

[18] Liang R., Yin T., Qin W. (2015). A simple approach for fabricating solid-contact ion-selective electrodes using nanomaterials as transducers. Anal. Chim. Acta.

[19] Ghosh T., Chung H.J., Rieger J. (2017). All-Solid-State Sodium-Selective Electrode with a Solid Contact of Chitosan/Prussian Blue Nanocomposite. Sensors.

[20] Arada-Perez M.A., Yazdani-Pedram M. (2013). Behavior of a polymeric liquid membrane ion- selective electrode for chloride ion. J. Chil. Chem. Soc..

[21] Arada-Perez M.A., Yanes S.L., Cardona M., Aguilera L., Yazdani-Pedram M. (2010). CopperII-Selective Electrodes based on 1-furoyl-3,3-diethylthiourea as a Neutral Carrier. J. Chil. Chem. Soc..

[22] Anastasova-Ivanova S., Mattinen U., Radu A., Bobacka J., Lewenstam A., Migdalski J., Danielewski M., Diamond D. (2010). Development of miniature all-solid-state potentiometric sensing system. Sens. Actuators B.

[23] Ngoc Mai P.T., Hoa P.T. (2015). Fabrication of Solid Contact Ion Selective Electrode for Mercury (II) Using Conductive Polymer Membrane. Mater. Trans..

[24] Rounaghi G., Kakhki R.M. (2011). Highly Selective and Sensitive Coated-Wire Yttrium (III) Cation Selective Electrode Based on Kryptofix-22DD. J. Electrochem. Soc..

[25] Yu S., Yuan Q., Li F., Liu Y. (2012). Improved potentiometric response of all-solid-state Pb2þ-selective electrode. Talanta.

[26] Yu S., Ju L., Xiong T., Yancang L., Liu Y. (2015). Solid-contact POlymeric Membrane electrode for Real-Time Monitoring of Lead Asorption. Int. J. Electrochem. Sci..

[27] Jaworska E., Schmidt M., Scarpa G., Maksymiuk K., Michalska A. (2014). Spray-coated all-solid-state potentiometric sensors. Analyst.

[28] Arada-Perez M.A., Marin L.P., Quintana J.C., Yazdani-Pedram M. (2003). Influence of different plasticizers on the response of chemical sensors based on polymeric membranes for nitrate ion determination. Sens. Actuators B.

[29] Telting-Diaz M., Bakker E. (2001). Effect of Lipophilic Ion-Exchanger Leaching on the Detection Limit of Carrier-Based Ion-Selective Electrodes. Anal. Chem..

[30] Cullen W., Turega S., Hunter C.A., Ward M.D. (2015). pH-dependent binding of guests in the cavity of a polyhedral coordination cage: Reversible uptake and release of drug molecules. Chem. Sci..

[31] Song Y., Ma L. (2015). A Solid-contact Pb 2+ -selective Electrode with Carbon Nanotubes by Electrodeposition as Ion-to-electron Transducer. Electrochemistry.

[32] Dai H., Wang N., Wang D., Ma H., Lin M. (2016). An electrochemical sensor based on phytic acid functionalized polypyrrole/graphene oxide nanocomposites for simultaneous determination of Cd(II) and Pb(II). Chem. Eng. J..

[33] Liu Y., Gao Y., Yan R., Huang H., Wang P. (2019). Disposable Multi-Walled Carbon Nanotubes-Based Plasticizer-Free Solid-Contact Pb 2+ Selective Electrodes with a Sub-PPB Detection Limit. Sensors.

[34] Upadhyay A., Singh A.K., Bandi K.R., Jain A.K. (2013). Fabrication of coated graphite electrode for the selective determination of europium (III) ions. Talanta.

[35] McCreery R.L. (2008). Advanced Carbon Electrode Materials for Molecular Electrochemistry. Chem. Rev..

[36] Svancara I., Walcarius A., Kalcher K., Vytras K. (2009). Carbon paste electrodes in the new millennium. Cent. Eur. J. Chem..

[37] Gooding J.J. (2005). Nanostructuring electrodes with carbon nanotubes: A review on electrochemistry and applications for sensing. Electrochim. Acta.

[38] Jacobs C.B., Peairs M.J., Venton B.J. (2010). Review: Carbon nanotube based electrochemical sensors for biomolecules. Anal. Chim. Acta.

[39] Ensafi A.A., Karimi-Maleh H. (2010). Modified multiwall carbon nanotubes paste electrode as a sensor for simultaneous determination of 6-thioguanine and folic acid using ferrocenedicarboxylic acid as a mediator. J. Electroanal. Chem..

[40] Khani H., Rofouei M.K., Arab P., Gupta V.K., Vafaei Z. (2010). Multi-walled carbon nanotubes-ionic liquid-carbon paste electrode as a super selectivity sensor—Application to potentiometric monitoring of mercury ion(II). J. Hazard. Mater..

[41] Tavana T., Khalilzadeh M.A., Karimi-Maleh H., Ensafi A.A., Beitollahi H., Zareyee D. (2012). Sensitive voltammetric determination of epinephrine in the presence of acetaminophen at a novel ionic liquid modified carbon nanotubes paste electrode. J. Mol. Liq..

[42] Huang J., Liu Y., Hou H., You T. (2008). Simultaneous electrochemical determination of dopamine, uric acid and ascorbic acid using palladium nanoparticle-loaded carbon nanofibers modified electrode. Biosens. Bioelectron..

[43] Wang C., Du J., Wang H., Zou C., Jiang F., Yang P., Du Y. (2014). A facile electrochemical sensor based on reduced graphene oxide and Au nanoplates modified glassy carbon electrode for simultaneous detection of ascorbic acid, dopamine and uric acid. Sens. Actuators B.

[44] Radhakrishnan S., Krishnamoorthy K., Sekar C., Wilson J., Kim S.J. (2014). A highly sensitive electrochemical sensor for nitrite detection based on Fe_2_O_3_ nanoparticles decorated reduced graphene oxide nanosheets. Appl. Catal. B: Environ..

[45] Du H., Ye J., Zhang J., Huang X., Yu C. (2011). A voltammetric sensor based on graphene-modified electrode for simultaneous determination of catechol and hydroquinone. J. Electroanal. Chem..

[46] Tan F., Cong L., Li X., Zhao Q., Zhao H., Quan X., Chen J. (2016). An electrochemical sensor based on molecularly imprinted polypyrrole/graphene quantum dots composite for detection of bisphenol A in water samples. Sens. Actuators B.

[47] Khasanah M., Handajana U.S., Widati A.A., Abdulloh A., Rindarti R.R. (2018). Construction and Performance of Creatinine Selective Electrode based on Carbon Paste-Imprinting Zeolite. Anal. Bioanal. Electrochem..

[48] Zou C., Yang B., Bin D., Wang J., Li S., Yang P., Wang C., Shiraishi Y., Du Y. (2017). Electrochemical synthesis of gold nanoparticles decorated flower-like graphene for high sensitivity detection of nitrite. J. Colloid Interface Sci..

[49] Ping J., Wu J., Wang Y., Ying Y. (2012). Simultaneous determination of ascorbic acid, dopamine and uric acid using high-performance screen-printed graphene electrode. Biosens. Bioelectron..

[50] Shao Y., Wang J., Wu H., Liu J., Aksay I.A., Lin Y. (2009). Graphene Based Electrochemical Sensors and Biosensors: A Review. Electroanalysis.

[51] Jawaheer S., White S.F., Rughooputh S.D.D.V., Cullen D.C. (2002). Enzyme stabilization using pectin as a novel entrapment matrix in biosensors. Anal. Lett..

[52] Willner I., Katz E., Willner B. (1997). Electrical Contact of Redox Enzyme Layers Associated with electrodes: Routes to Amperometric Biosensors. Electroanalysis.

[53] Karube I., Nomura Y. (2000). Enzyme sensors for environmental analysis. J. Mol. Catal. B Enzym..

[54] Jomma E.Y., Ding S. (2016). Recent Advances on Electrochemical Enzyme. Biosensors.

[55] Erden P.E., Pekyarimci S., Kilic E. (2012). Amperometric Enzyme Electrodes for Xanthine Determination with Different Mediators. Acta Chim. Slov..

[56] Achmann S., Hammerle M., Moos R. (2008). Amperometric enzyme-based biosensor for direct detection of formaldehyde in the gas phase: Dependence on electrolyte composition. Electroanalysis.

[57] Suprun E.V., Budnikov H.C., Evtugyn G.A., Brainina K.Z. (2004). Bi-enzyme sensor based on thick-film carbon electrode modified with electropolymerized tyramine. Bioelectrochemistry.

[58] Bhattachayay D., Pal P., Banerjee S., Sanyal S.K., Turner A.P.F., Sarkar P. (2008). Electrochemical acetylcholine chloride biosensor using an acetylcholine esterase biomimic. Anal. Lett..

[59] Apetrei I.M., Rodgriguez-Mendez M.L., Apetrei C., de Saja J.A. (2018). Enzyme sensor based on carbon nanotubes/cobalt(II) phthalocyanine and tyrosinase used in pharmaceutical analysis. Sens. Actuators B.

[60] Kudo H., Yagi T., Chu M.X., Saito H., Morimoto N., Iwasaki Y., Akiyoshi K., Mitsubayashi K. (2008). Glucose sensor using a phospholipid polymer-based enzyme immobilization method. Anal. Bioanal. Chem..

[61] Hernandez-Cazares A.S., Aristoy M., Toldra F. (2010). Hypoxanthine-based enzymatic sensor for determination of pork meat freshness. Food Chem..

[62] Ajay A.K., Srivastava D.N. (2007). Microtubular conductometric biosensor for ethanol detection. Biosens. Bioelectron..

[63] Chuang C., Shih J. (2001). Preparation and application of immobilized C60-glucose oxidase enzyme in fullerene C60-coated piezoelectric quartz crystal glucose sensor. Sens. Actuators B.

[64] Kobayashi Y., Anzai J. (2001). Preparation and optimization of bienzyme multilayer films using lectin and glyco-enzymes for biosensor applications. J. Electrochem..

[65] Yabuki S., Hirata Y., Sato Y., Iijima S. (2012). Preparation of a Cellulose-based Enzyme Membrane Using Ionic Liquid to Lengthen the Duration of Enzyme Stability. Anal. Sci..

[66] Ammam M., Fransaer J. (2010). Two-enzyme lactose biosensor based on beta-galactosidase and glucose oxidase deposited by AC-electrophoresis- Characteristics and performance for lactose determination in milk. Sens. Actuators B.

[67] Siqueira J.R., Molinnus D., Beging S., Schoning M.J. (2014). Incorporating a Hybrid Urease-Carbon Nanotubes Sensitive Nanofilm on Capacitive Field-Effect Sensors for Urea Detection. Anal. Chem..

[68] Liu H., Deng J. (1995). An Amperometric lactate Sensor Employing Tetrathiafulvalene in Nafion Film as Electron Shuttle. Electrochim. Acta.

[69] Mocak J., Bond A.M., Mitchell S., Scollary G. (1997). A statistical overview of standard (iupac and acs) and new procedures for determining the limits of detection and quantification: Application to voltammetric and stripping techniques. Pure Appl. Chem..

[70] Perone S.P., Kretlow W.J. (1965). Anodic Stripping Voltammetry of Mercury(II) at the Graphite Electrode. Anal. Chem..

[71] Watson C.M., Dwyer D.J., Andle J.C., Bruce A.E., Bruce M.R.M. (1999). Stripping Analyses of Mercury Using Gold Electrodes: Irreversible Adsorption of Mercury. Anal. Chem..

[72] Forsberg G., O’Laughlin J.W., Megargle R.G., Koirtyohann S.R. (1975). Determination of Arsenic by Anodic Stripping Voltammetry and differential Pulse Anodic Stripping Voltammetry. Anal. Chem..

[73] Boukoureshtlieva R.I., Hristov S.M., Milusheva Y.D., Atanassov P.B., Kaisheva A.R. (2011). Mediated enzyme electrodes. Bulg. Chem. Commun..

[74] Yu E.H., Sundmacher K. (2007). Enzyme electrodes for glucose oxication prepared by electropolymerization of pyrrole. Process. Saf. Environ. Prot..

[75] Shen L., Chen Z., Li Y., He S., Xie S., Xu X., Liang Z., Meng X., Li Q., Zhu Z. (2008). Electrochemical DNAzyme Sensor for Lead Based on Amplification of DNA-Au Bio-Bar Codes. Anal. Chem..

[76] McMillan D.G.G., Marritt S.J., Kemp G.L., Gordon-Brown P., Butt J.N., Jeuken L.J.C. (2013). The impact of enzyme orientation and electrode topology on the catalytic activity of adsorbed redox enzymes. Electrochim. Acta.

